# T-cell lymphoma infiltrating the uterus and ovaries of a Golden Retriever: a case report

**DOI:** 10.1186/s13620-023-00252-x

**Published:** 2023-09-13

**Authors:** Jaeyeop Jo, Mingyun Son, Yeon Chae, Taesik Yun, Yoonhoi Koo, Dohee Lee, Hyun-Gu Kang, Byeong-Teck Kang, Mhan-Pyo Yang, Hakhyun Kim

**Affiliations:** 1 Laboratory of Veterinary Internal Medicine, College of Veterinary Medicine, Chungbuk National University, Cheongju, 28644 Chungbuk Republic of Korea; 2 Present Address: College of Veterinary Medicine, Kyungpook National University, Daegu, 41566 Republic of Korea; 3 Laboratory of Veterinary Obstetrics, College of Veterinary Medicine, Chungbuk National University, Cheongju, 28644 Republic of Korea

**Keywords:** Canine, Genital disease, Lymphoma, Ovariohysterectomy, Pyometra

## Abstract

**Background:**

To the best of our knowledge, this is the first report of female genital system infiltration of T-cell lymphoma in veterinary literature.

**Case presentation:**

A 1.5-year-old, intact female Golden Retriever was referred due to melena and hyporexia that lasted for three weeks. Fever (40.5℃), tachycardia, tachypnoea, pale mucous membranes, and purulent vaginal discharge were identified on physical examination. Blood analyses revealed leucocytosis, anaemia, hypoalbuminemia, and increased lactate and C-reactive protein levels. On abdominal radiography, the small intestine was moderately deviated because of an oval-shaped mass (13 cm × 8.7 cm) located in the mid-abdomen. An enlarged tubular-shaped structure that had the opacity of soft tissue located in dorsal to the bladder to the middle of the abdomen, and an oval-shaped mass (5.28 cm × 3.26 cm), which was suspected to be a medial iliac lymph node located at the sixth to seventh lumbar level. Abdominal ultrasonography revealed gas and fluid in the lumen of the uterine horn with a severely thickened wall, round enlarged lymph nodes around the genitourinary system, and free fluid in the abdominal cavity. Based on these results, pyometra was suspected, and an exploratory laparotomy was performed for ovariohysterectomy. The resected ovary and uterus were macroscopically hypertrophied. Histopathological examination of the ovary and uterus revealed neoplastic proliferation of large round cells with strong immunoreactivity for CD3, indicating T-cell lymphoma. Therefore, the young dog was diagnosed with genital lymphoma.

**Conclusions:**

The present report describes T-cell lymphoma infiltrating the uterus and ovaries in a young dog, which is rarely diagnosed and could aid in the differential diagnosis of genital diseases in young dogs.

## Background

Canine lymphoma is classified according to anatomic site: multicentric (80% of cases), alimentary (7% of cases), cutaneous (6% of cases), mediastinal (3% of cases), and miscellaneous extranodal sites (1% of cases) [1–3]. Canine lymphoma mainly occurs at a median age of 6–9 years and has a low prevalence rate in intact females [4]. Therefore, extranodal lymphoma affecting the female genital tract is rare, especially in young dogs.

There are three classes of female reproductive system tumours: ovarian, uterine, and vaginal/vulvar. The prevalence of ovarian, uterine, and vaginal/vulvar tumours is 0.5–1.2%, 0.3–0.4%, and 2.4-3%, respectively [5–10]. Among canine female reproductive system tumours, epithelial tumours (adenoma, adenocarcinoma) and leiomyomas are the most common, whereas lymphoma is rare [5, 9–17].

This case report describes the clinical signs, laboratory findings, imaging features, and histological characteristics of peripheral T-cell lymphoma infiltrating the female genital system in a young dog, which is extremely rare and could be considered as a differential diagnosis of genital disease in dogs.

## Case presentation

A 1.5-year-old intact female Golden Retriever weighing 25 kg presented with melena and hyporexia that had lasted for three weeks. There was no history of medication, or toxin or foreign body ingestion. The dog was regularly vaccinated for canine distemper virus, infectious canine hepatitis, parainfluenza virus, canine parvovirus and heart worm disease. The owner observed estrus approximately 5 months prior to presentation, but was unable to recall if it was the most recent one. The dog’s vitality had gradually deteriorated over time. The dog drank water well, but it was uncertain if there was polydipsia and polyuria present. On physical examination, the dog showed fever (rectal temperature, 40.5℃), tachycardia (heart rate, 180 beats per minute), normotensive (systolic blood pressure, 136 mmHg), and tachypnoea (respiratory rate, 80 breaths per minute). Her mucous membranes were pale and tacky, and sunken eyes were observed. All mammary glands were swollen and pliable, but not painful. Generalized abdominal pain was observed on palpation. The vulva was mildly swollen and accompanied by purulent vaginal discharge with a foul odour.

Complete blood counts of the dog revealed leucocytosis with a left shift and regenerative anaemia (Table 1). Abnormalities in serum biochemistry profile included decreased serum total protein, albumin, globulin and glucose levels and increased serum blood urea nitrogen, symmetric dimethylarginine, serum lactate and C-reactive protein levels. Additionally, serum aspartate aminotransferase, alanine aminotransferase and alkaline phosphatase activities were increased. Abnormalities in serum electrolyte profile included mildly decreased concentrations of sodium and total calcium, as well as an increased phosphorus concentration.

**Table 1:** Results of blood analyses of the present case

Parameters	Values	Reference intervals
Whole blood counts (×10^3^/µL)	31.70	5.05–16.76
Neutrophils (×10^3^/µL)	25.23	2.95–11.64
Monocytes (×10^3^/µL)	3.04	0.16–1.12
Lymphocytes (×10^3^/µL)	3.28	1.05–5.10
Eosinophils (×10^3^/µL)	0.03	0.06–1.23
Basophils (×10^3^/µL)	0.12	0–0.10
Packed cell volume (%)	10.8	37.3–61.7
Reticulocyte (×10^3^/µL)	445.4	10.0–110.0
Platelet (×10^6^/µL)	381	148–484
Total protein (g/dL)	4.4	5.4–7.1
Albumin (g/dL)	2.2	2.6–3.3
Globumin (g/dL)	2.2	2.7–4.4
AST (IU/L)	1003	23–66
ALT (IU/L)	2059	21–102
GGT (IU/L)	5	1–10
ALP (IU/L)	424	29–97
Bile acid (µmol/L)	0	0–25
Total bilirubin (mg/dL)	0.2	0.1–0.5
Blood urea nitrogen (mg/dL)	31.8	7–25
Creatinine (mg/dL)	0.8	0.5–1.5
SDMA (µg/dL)	31	0–14
Total cholesterol (mg/dL)	139	135–270
Triglyceride (mg/dL)	59	21–116
Glucose (mg/dL)	62	65–118
CPK (IU/L)	467	42–530
Lactate (mmol/L)	> 12.0	0.5–2.5
CRP (mg/L)	108.16	0–10
Sodium (mmol/L)	140	141–152
Potassium (mmol/L)	5.8	3.6–5.8
Chloride (mmol/L)	108	105–115
Total calcium (mg/dL)	8.8	9–11.3
Phosphorus (mg/dL)	6.7	2.6–6.2

*ALT *Alanine aminotransferase, *AST *Aspartate aminotransferase, *ALP *Alkaline phosphatase, *BUN *Blood urea nitrogen, *CPK *Creatinine phosphokinase, *CRP *C-reactive protein,  *GGT *Gamma-glutamyl transferase, *SDMA *Symmetric dimethylarginine

**Fig. 1:**
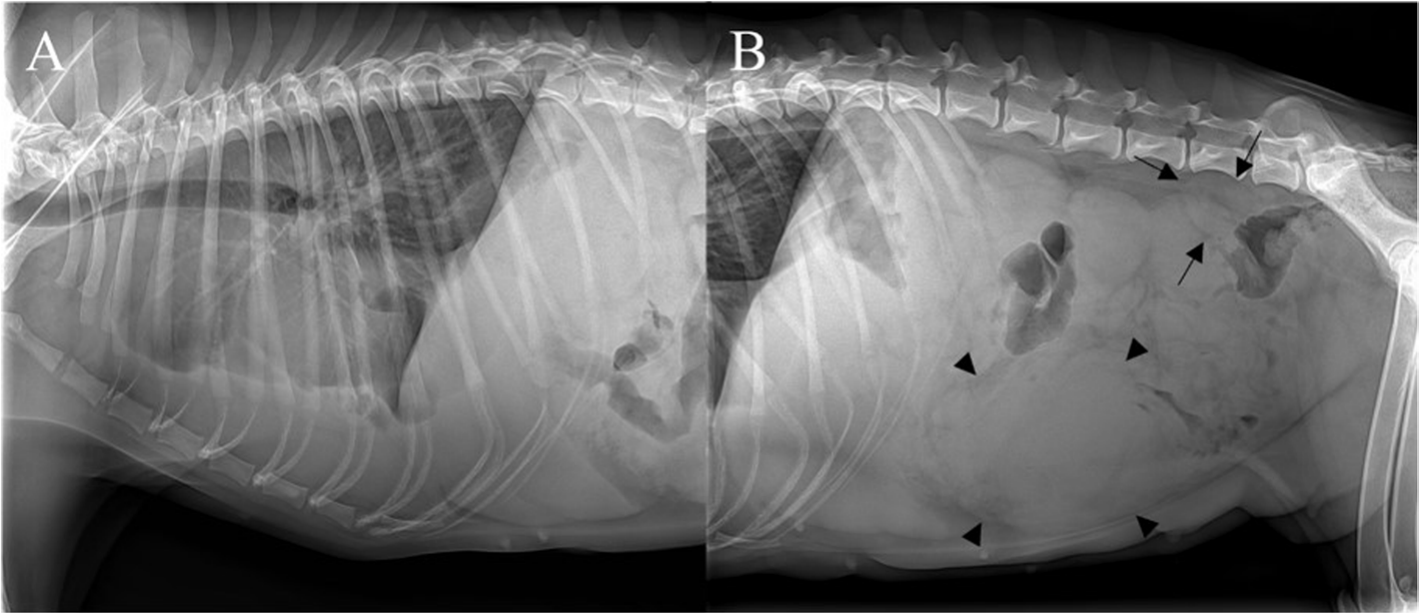
Radiographic findings. **A** Right lateral view of the thorax radiography revealing interlobar fissure and obscuring visualization of the cardiac silhouette. **B** Right lateral view of the abdominal radiography revealing low serosal detail, an oval-shaped mass (arrowhead, 13 × 8.7 cm) located inter-mid of the abdomen, and an oval-shaped mass (arrow, 5.28 × 3.26 cm) located in the sixth to seventh lumbar level

Radiography revealed an interlobar fissure and obscuring visualization of the cardiac silhouette in the thorax, indicating pleural effusion, and low serosal detail was revealed in the abdomen, indicating suspected abdominal effusion. Furthermore, the small intestine was moderately deviated because of an oval-shaped mass (13 cm × 8.7 cm) located in the middle of the abdomen. Moreover, an enlarged tubular-shaped structure that had the opacity of soft tissue was located in the dorsal to the bladder to the middle of the abdomen and an oval-shaped mass (5.28 cm × 3.26 cm), which was suspected to be a medial iliac lymph node, was located in the sixth to seventh lumbar level (Fig. 1). Abdominal ultrasonography was performed to assess these abdominal cavity abnormalities.

**Fig. 2:**
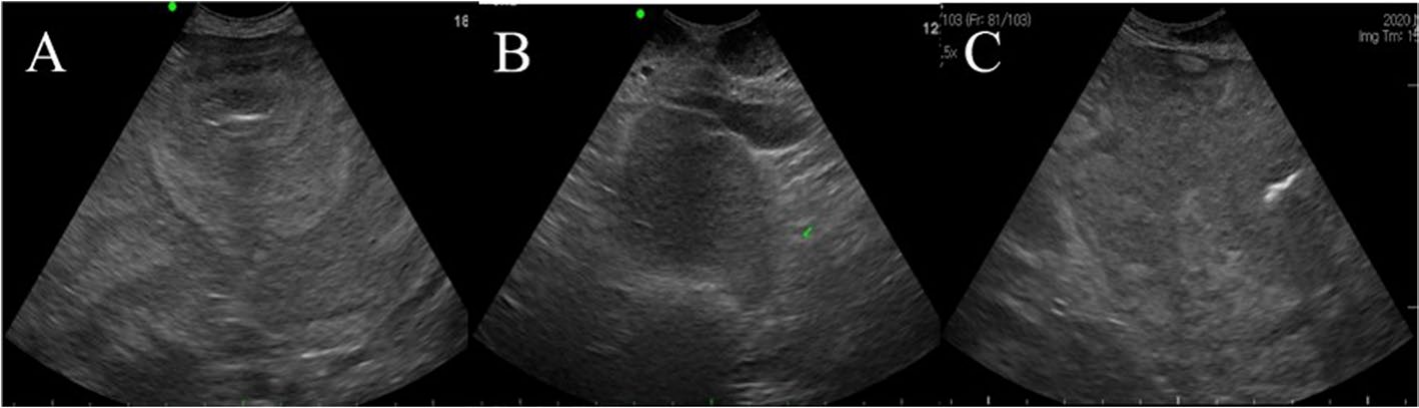
Abdominal ultrasonographic findings. **A** Muscular layer thickening of the gastrointestinal tract wall is detected. **B** Free fluid, hyperechoic mesenteric fat, and mesenteric lymph node enlargement are detected (arrow). **C** Gas and fluid are identified in the uterine lumen

On ultrasonography, some segments of the muscular layers of the small and large intestine were thickened (> 0.9 and 0.7 mm, respectively). A moderate amount of free fluid and hyperechoic mesenteric fat were also observed in the abdominal cavity. This echogenic free fluid was located around the uterine horn. Gas and fluid were detected in the lumen of the uterine horn with a severely thickened wall, and enlarged round lymph nodes were identified around the genitourinary system (Fig. 2).

**Fig. 3:**
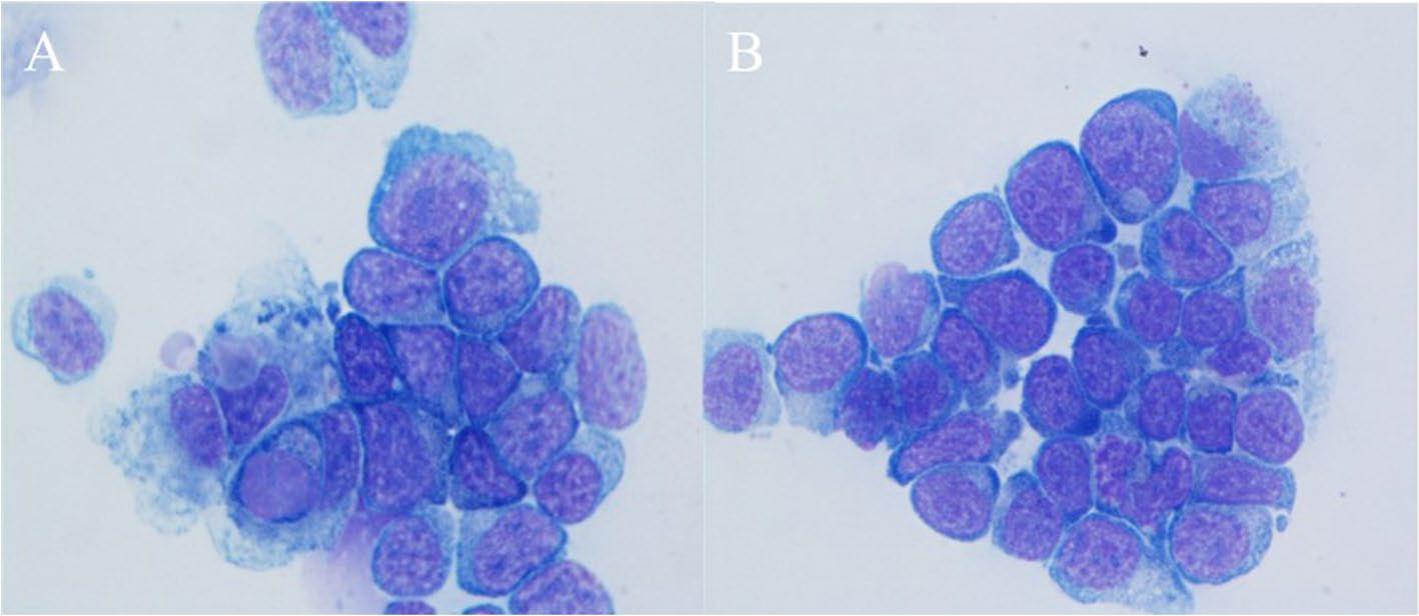
Cytologic findings of the thoracocentesis and abdominocentesis for pleural and peritoneal fluids, respectively. **A** Pleural effusion with predominant lymphocytes. **B** Ascites with predominant lymphocytes are identified

Thoracocentesis was performed at the right 7th intercostal space to relieve the pleural effusion and differentiate the cause of the effusion, and abdominocentesis was performed at the right caudal quadrant of the abdomen to differentiate the cause of the ascites. The pleural effusion type was modified transudate (total protein, 1.5 g/dL; total nuclear cells, 4,670 cells/µL) which had predominant lymphocytes, whereas the ascites type was non-septic exudate (total protein, 1.8 g/dL; total nuclear cells, 10,350 cells/µL) that also had predominant lymphocytes (Fig. 3). In both effusions, 90% of the nucleated cells were lymphocytes, and the nuclei of the lymphocytes were larger than two red blood cells [18] with finely stippled-to-clumped chromatin, and often eccentric. Nucleoli sizes also differed. Furthermore, the lymphocytes had basophilic cytoplasm. The cytological examination indicated a large number of lymphoblasts in both effusions.

Systemic inflammatory response syndrome (SIRS) was suspected based on pyrexia (40.5℃), tachycardia (heart rate, 180 beats per minute), tachypnoea (respiratory rate, 80 breaths per minute), and leucocytosis with a left shift, which met SIRS criteria [19] (at least two of the following four criteria need to be met: hypothermia [rectal body temperature < 38.1 ℃] or hyperthermia [rectal body temperature > 39.2 ℃]; heart rate > 120 beats per minute; respiratory rate > 20 breaths per minute; and leucopoenia (white blood cell count < 6.0 × 10^9^/L), leucocytosis (white blood cell count > 16.0 × 10^9^/L), or > 3% band neutrophils. Moreover, vaginal discharge, mass effect on the abdominal radiograph, gas and fluid in the lumen of the uterus, and severely thickened uterine wall were detected on ultrasonographic examinations. Therefore, pyometra was suspected, and exploratory laparotomy was performed for ovariohysterectomy to remove the uterus and both ovaries.

Before the operation, ampicillin-sulbactam (20 mg/kg, three times daily; Ubacillin; Whanin Pharm Co., Ltd., Seoul, South Korea) and butorphanol (0.2 mg/kg third times daily; Butophan; MyungMoon Pharm Co., Ltd., Seoul, South Korea) were administered intravenously. Enrofloxacin (5 mg/kg once daily; Baytril; Bayer Inc., Leverkusen, North Rhine-Westphalia, Germany) and maropitant citrate (1 mg/kg once daily; Cerenia; Pfizer Inc., New York City, Manhattan, United States of America) were administered subcutaneously. Intravenous fluid therapy was administered to treat dehydration and pyrexia. Furthermore, to manage pyrexia fan directed at the dog and cold water was applied on the dog’s whole body. Approximately 600 ml of whole blood were also administered to manage anaemia 8 h prior to surgery. After the vital signs had stabilised for 8 h, exploratory laparotomy with midline incision and surgical resection of the uterus were performed to treat and diagnose the uterine abnormalities. During surgery, mild pinkish fluid throughout the entire abdomen and adhesion of uterine and ovaries were identified. No obvious abnormalities were noted in the liver, spleen, stomach, pancreas, kidney, and urinary bladder. However, the adrenal glands were not identified. Swelling and redness were noted in some segments of small and large intestines. The lymph nodes around the ovaries and uterine were enlarged. Resected ovarian and uterine tissues were fixed in 10% neutral buffered formalin and submitted for histopathological examination. Following the ovariohysterectomy, the dog’s vital signs deteriorated during intensive care. The dog exhibited persisted tachycardia, tachypnoea, and hypotension. Additionally, the dog displayed a depressed mental status. Subsequently, euthanasia was performed with the consent of the owner based on the concern in terms of the guarded prognosis, and in consideration of the dog’s post-operative condition.

**Fig. 4:**
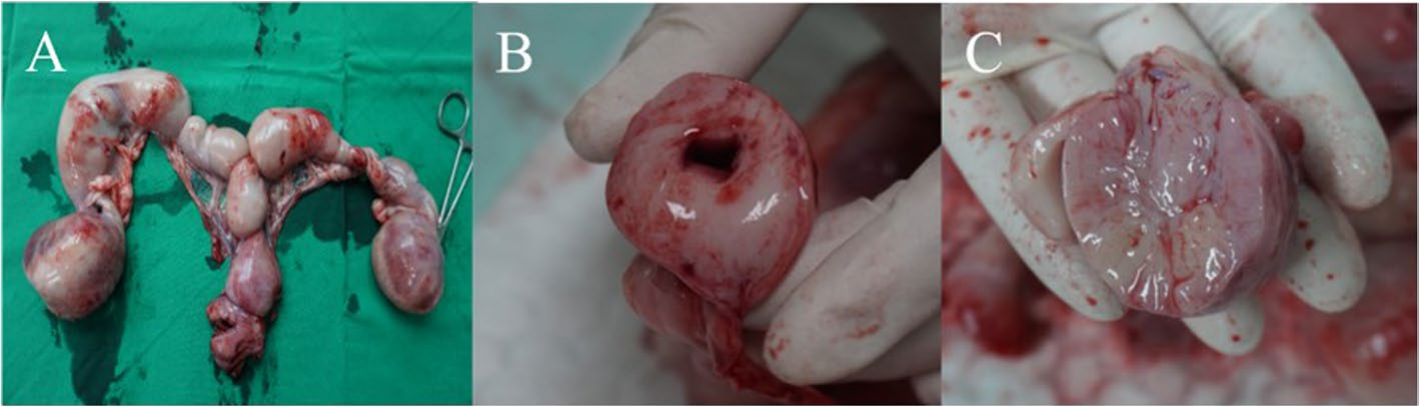
Gross findings. **A** Pale and various sized multiple masses in the ovary and uterus are observed. **B** Cross section of the uterine horn reveals pale and homogenous colour and solid on palpation. **C** Cross section of the ovary reveals pale and heterogeneous colour and soft on palpation

**Fig. 5:**
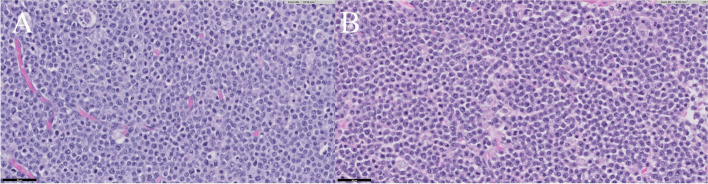
Histopathologic findings of the ovaries and uterus. **A** The section of ovarian cortex is completely effaced by sheets of neoplastic round cells consistent with lymphocytes (H&E. Bar = 50 μm). These cells have scant cytoplasm with a round nucleus containing coarse chromatin and variably distinct rounded nucleoli. **B** Morphologically similar characteristics of neoplastic round cells in the ovaries are also observed in the uterus. (H&E. Bar = 50 μm)

**Fig. 6:**
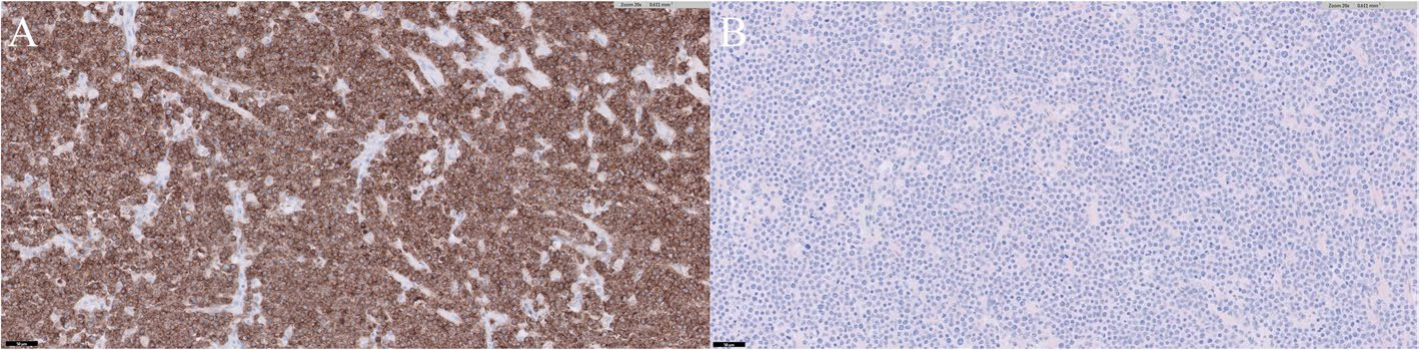
Results of immunohistochemistry. **A** Diffused neoplastic cells showing strong positive immunoreactivity (CD3; Bar = 50 μm). **B** Diffused neoplastic cells showing negative immunoreactivity (PAX5; Bar = 50 μm)

On gross appearance, multiple masses were observed in the ovary and uterus (Fig. 4). On sectioning both ovary and uterus were markedly enlarged. Furthermore, the cross sections of uterine were filled with firm masses with mild purulent fluid. Histopathological examination of the resected ovaries showed that round cells had invaded and destroyed the muscularis layer and invaded the attached mesenteric fat. The cells were approximately twice the size of an erythrocyte. The mitotic count was 35 per 10 high-power fields and vascular and lymphatic invasion were detected. Similar characteristics were observed in both ovaries. Finally, large-cell lymphoma was confirmed in both ovaries (Fig. 5A). Neoplastic proliferation of large round cells was observed with small number of inflammatory cells such as neutrophils and macrophages in the uterus. Unlike in the ovary, the mitotic count was 42 per 10 high power fields and vascular and lymphatic invasion were not detected. Finally, large-cell lymphoma was confirmed to be the same as that of the ovaries (Fig. 5B). In both the ovaries and uterus, diffused neoplastic cells had strong positive immunoreactivity for CD3 and negative immunoreactivity for PAX5 in immunohistochemistry (Fig. 6). Based on these results, the patient was diagnosed with peripheral T-cell lymphoma infiltrating the female genital system.

## Discussion and conclusions

Generally, many clinicians consider pyometra in female dogs to present with acute hyporexia, fever, abdominal pain, and vaginal discharge. Similarly, pyometra was initially suspected in the present case because of the SIRS-compliant status, vaginal discharge, mass effect of the abdominal radiograph, gas and fluid in the lumen of the uterus, and severely thickened uterine wall. However, the present case was a relatively young. Pyometra generally occurs in middle-aged and older female dogs, although it has been reported in age groups ranging from 9 months to 18 years [20], which are similar to the prevalent age of the canine lymphoma [4]. Therefore, the age of presentation could not be used to differentiate between pyometra and lymphoma in our case. Finally, the dog was diagnosed with T-cell lymphoma involving the ovaries and uterus. As a result, a mass effect observed in the middle of the abdomen and macroscopic hypertrophy of the genital system were identified as multiple lymphomas through histopathologic examination. Immunohistochemistry revealed strong positive immunoreactivity for CD3 and negative immunoreactivity for PAX5. CD3 is an immune-mediated marker of T-cell lymphoma and PAX5 is an immune-mediated marker of B-cell lymphoma. In veterinary medicine, canine lymphomas of B-cell origin account for 60–80% of cases, T-cell origin accounts for 10–38% of cases, mixed B- and T-cell constitute approximately 22% of cases, and neither B- or T-cell origin account for less than 5% of cases [3]. Finally, to the best of our knowledge, this is the first report of infiltration of T-cell lymphoma into the female genital system in veterinary literature. However, we could not completely exclude a concurrent pyometra which could deteriorate the present dog’s vitality after surgery because we did not perform the examination of the luminal contents of the uterus.

Metastasis from a possible primary tumour in the ovaries was suspected because vascular and lymphatic invasion were identified on histopathological examination of the gonads. However, this suspicion remains speculative as a full post-mortem examination was not performed. In this case, because the origin of the lymphoma was ambiguous, we considered two scenarios. The first scenario is primary T-cell lymphoma of the extranodal sites. Only one report has described primary lymphoma of the female genital system in veterinary medicine. This report presents a case of extranodal mucosa-associated lymphoid tissue (MALT)-type marginal zone B-cell lymphoma in the uterus [21]. The uterine mucosa was composed of MALT, a particular form of peripheral lymphoid tissue. Marginal zone B-cell lymphoma, an extranodal type, can originate from MALT within various sites, including the orbit, thyroid, salivary glands, lungs, intestines, and uterus [22]. To the best of our knowledge, our case is the first report of T-cell-type lymphoma.

The second scenario is metastasis of gastrointestinal (GI) lymphoma to the genital system. Generally, canine GI lymphoma is characterized by nonspecific GI signs such as malabsorption, weight loss, diarrhoea, and vomiting [23–25]. In addition, canine GI lymphoma may arise focally but more often affects multiple segments, with narrowing of the lumen, wall thickening, and frequent mucosal ulceration [23, 26]. Furthermore, according to a recent study, primary GI lymphoma of T-cell origin is more common than that of B-cell origin [27]. In this case, melena lasted for three weeks, the muscular layer of some intestinal segments was thickened, and the mesenteric fat was hyperechoic. However, these signs are not specific to GI lymphoma, and other intestinal disorders may also show these signs. Nevertheless, we could not completely exclude either scenario as we did not undertake an intestinal biopsy during exploratory laparotomy or a post-mortem examination to determine where the T-cell lymphoma had primarily originated: i.e. whether there had been metastasis of primary GI T-cell lymphoma to the genital tract or primary genital tract T-cell lymphoma to the GI tract.

Despite intensive care, the dog’s condition was progressively deteriorated following surgical resection of the ovarian and uterine lymphoma. Specific reasons for this deterioration are not known as the owner’s declined any further work-up to assess the dog’s status. Given the vital signs and blood test results at presentation, we considered SIRS to be one possible reason for the further post-operative deterioration. SIRS describes a clinical condition with widespread inflammation, secondary to infectious or sterile inflammatory disease [28]. SIRS could have occurred either as a consequence of uterine malignancy or of malignant lymphoma itself in this case. SIRS has a role in the initiation, promotion, and progression stages of the neoplastic process [29]. In addition, SIRS is an important indicator of survival in various female genital tract malignancies in humans [30–32]. Neoplastic cells in the genital tract can secrete various cytokines including interleukin-6, which then recruit inflammatory cells [33, 34]. Furthermore, serum levels of interleukin-6 have been shown to be significantly higher in dogs with multicentric T-cell lymphoma than in those with B-cell lymphoma and healthy dogs [35]. Interleukin-6, a pleiotropic immunomodulatory cytokine produced by malignant T-cell lymphocytes, promotes inflammation through the expansion and activation of T-cells, differentiation of B-cells, and the induction of acute-phase proteins by hepatocytes [36]. Therefore, many pro-inflammatory cytokines are produced in this process along with malignant lymphocytes, which induce systemic inflammation when pro-inflammatory cytokines overwhelm anti-inflammatory cytokines. Indeed, an elevated pre-treatment neutrophil-to-lymphocyte ratio, a marker for SIRS, has prognostic significance in humans with ovarian cancer and T-cell lymphoma [37, 38]. Therefore, we assumed that the cause of the deterioration might have been due to further progression of SIRS in the present case after the surgery despite having stabilized the dog’s vital signs prior to surgical resection of ovarian and uterine lymphoma. However, further research is needed to clarify the prognostic significance of pre-treatment SIRS in dogs with genital tract lymphoma. Furthermore, there was a possibility of the bacterial systemic infection based on the purulent vaginal discharge and gas and fluid accumulation in the uterine lumen indicating concurrent pyometra. Moreover, pyometra could be occurred due to the impaired local innate immune system due to the uterine lymphoma. Therefore, the cause of the deterioration might also have been bacterial systemic infection and toxaemia, even though broad-spectrum antibiotics and intravenous fluid administered. Unfortunately, we did not culture the luminal contents of the uterus. Therefore, we did not elucidate whether the cause of the further deterioration of the present case was due to pre-treatment SIRS related with lymphoma, bacterial systemic infection, or both.

Malignant lymphocytes in both the pleural and abdominal effusions might be another possible cause of the poor prognosis in the present case. The cytologic examination showed a large number of lymphoblasts in both effusion, indicating that T-cell lymphoma had also infiltrated the pleura and peritoneum. The infiltrated lymphoma could have increased the permeability of the capillaries in the pleura and peritoneum, which then facilitated the entry of proteins into the thoracic and abdominal cavities, leading to effusions in both cavities. This scenario might explain the characteristics of the both effusion (modified transudate and non-septic exudate). Therefore, it is possible that systemic infiltration of T-cell lymphoma led to the dog’s deteriorating vital signs post-operatively. The presence of pleural effusion might reduce survival time in dogs with high-grade mediastinal lymphoma owing to the tumour’s aggressive characteristics [18]. However, pleural and peritoneal biopsies would have been necessary to confirm this scenario as malignant lymphocytes are difficult to distinguish from reactive lymphoid cells in effusions [39] although the lymphocytes in the both effusions had malignant features in the present case.

In conclusion, a diagnosis of T-cell lymphoma involving the ovaries and uterus was reached, a very unlikely occurrence in such a young dog. Even in situations that comply with SIRS and vaginal discharge is identified, lymphoma should also be considered as a differential diagnosis if it is not typical pyometra in dogs.

## Data Availability

Not applicable.

## Abbreviations

GI: Gastrointestinal
MALT: Mucosa-associated lymphoid tissue
SIRS: Systemic inflammatory response syndrome
